# Disease burden, treatment experiences and preferences in patients with acromegaly: a qualitative study

**DOI:** 10.3389/fendo.2026.1733510

**Published:** 2026-03-05

**Authors:** Jennifer Quinn, Andrea De Palma, Rebecca McKeown, Rocco Adiutori, Charlotte E. Kosmas, Isabelle Petit

**Affiliations:** 1 Global Value & Access, Debiopharm International SA, Lausanne, Switzerland; 2 Erasmus School of Health Policy & Management, Erasmus Rotterdam University, Rotterdam, Netherlands; 3 ICON plc, Insights, Evidence and Value, Reading, United Kingdom; 4 Mapi Research Trust, Lyon, France; 5 ICON plc, Insights, Evidence and Value, Milan, Italy

**Keywords:** acromegaly, disease burden, somatostatins, treatment burden, treatment experience, treatment preference

## Abstract

**Purpose:**

Acromegaly is a rare disease with limited treatment options. Understanding treatment burden and patient preferences is important for evaluating new treatments and optimizing adherence.

**Methods:**

Patients with acromegaly (≥18 years) from the United States (US) participated in qualitative semi-structured interviews to explore: 1) the impact of acromegaly on quality of life, 2) patients’ experiences with current treatment, 3) preferences for new treatments.

**Results:**

Fifteen US patients with acromegaly participated, reporting a range of symptoms; physical changes/swelling, fatigue/tiredness and excessive sweating. Frequently reported impacts included limited socializing (n = 6), anxiety (n = 4), embarrassment due to sweating/odor (n = 4) and clothing adaptations due to swelling (n = 4). Frequently reported impacts associated with monthly injectable treatment included unpleasantness of injections/blood tests (n = 6), clinic waiting time (n = 5), travelling to the clinic (n = 3), and treatment frequency (n = 3). Over half of patients preferred daily oral treatment options (n = 8, 53.3%). When asked about preference around hypothetical treatment frequency, 60.0% (n=9) preferred a hypothetical 3-monthly injection compared to a monthly injection if it was as efficacious as the monthly injection or recommended by their doctor.

**Conclusion:**

Patients experience a wide range of symptoms and impacts, with a high burden of treatment associated with monthly injections. Patients demonstrated preferences for less frequent treatments, with a preference for reducing their current monthly injections to three or six monthly. When considering new treatments, the efficacy and safety profile were of most importance to patients with acromegaly.

## Introduction

Acromegaly is a rare disease in which the body produces excess growth hormone (GH) and insulin-like growth factor 1, causing tissues and bones to grow excessively (1). The disease presents with a variety of different symptoms, including abnormally large hands and feet, enlarged facial features, headaches, excessive perspiration, hypertension, oligomenorrhea, arthralgias, carpal tunnel syndrome, and type 2 diabetes mellitus (2, 3). Symptoms are gradual in presentation which leads to delay in diagnosis, with an average time of 5–10 years between onset and diagnosis (2, 3). The disease impacts patients’ lives at the emotional, social, daily, work, physical, and financial level (4–16). To reduce morbidity and normalize mortality, lifelong medical treatment is required, additionally impacting adherence and treatment experiences (5–8).

Somatostatin analogs (SSA) lanreotide (Somatuline) and octreotide (Sandostatin) are used to treat the symptoms of acromegaly by suppressing GH secretion. SSA are usually administered monthly through an intramuscular injection (1). Orally administered octreotide (Mycapssa) capsules taken twice daily have also become available (1).

To date, there is little published evidence on the burden of disease and treatment in patients with acromegaly, such as the burden associated with monthly injections or daily oral medication, or the preferences of patients regarding treatment. This study aimed to understand the lived experiences of patients living with acromegaly, including the disease symptoms, impacts and treatment burden. In addition, it aimed to assess patient preferences in relation to a sustained release treatment for this population in order to inform the development of new therapies.

## Methods

### Study design

A cross-sectional, qualitative study was designed, enrolling adults diagnosed with acromegaly or gastroenteropancreatic neuroendocrine tumors in the United States (US). In this article, only data from the patients with acromegaly (n = 15) are reported.

To support the development of the interview guide, a targeted literature review was conducted to identify qualitative studies which reported disease experience and treatment burden of patients with acromegaly, methods and results of which are found in the **Supplementary Material**. A semi-structured interview was developed based on literature review findings. Information about the articles found in the literature review and the interview guide featuring key sections, domains and example questions can be found in the **Supplementary Files**.

Patients underwent one-time individual, semi-structured, concept elicitation web-assisted interviews aimed to understand patient experience of acromegaly, their treatment and treatment preferences. Interviews lasted approximately 60 minutes. The qualitative research protocol and related study documents were reviewed and approved by Salus IRB on 9^th^ March 2023, for the conduct of the research in the US (protocol number 0186-0062).

### Sample and recruitment

Participants were recruited in this study using a specialist recruitment agency who identified patients through their patient databases, physician referrals, associations, patient advocacy groups, support groups and social media advertising. They used IRB approved recruitment materials to conduct this process. Eligibility criteria included adults (18 to 75 years old) with a diagnosis of acromegaly receiving octreotide or lanreotide (SSA). Patients self-reported their diagnosis but were required to answer screening questions to ascertain a current diagnosis of acromegaly. Patients taking octreotide capsules (Mycapssa; herein referred to as oral treatment), must have received injectable SSA (herein referred to as injectable treatment) in the past six months and been on a stable dose of injectable treatment for at least three months prior to making the change to oral treatment. Patients were reimbursed for their time ($150.00). As this was an exploratory qualitative interview study, no formal sample size calculation was conducted.

### Concept elicitation interviews

Trained and experienced interviewers conducted interviews between May 2023 and June 2023. Interviews were conducted using a web-based platform, recorded and transcribed.

Patients provided verbal informed consent to participate in the study at the beginning of the interview. During the interview, patients were asked questions about their condition, treatment, and preferences that affect the decisions they made about their treatment.

### Qualitative analysis

Thematic analysis (17) of de-identified transcripts were performed using MAXQDA software (18) to organize concepts that arose from the interviews. Transcripts were coded independently by two trained analysts who developed and updated the coding framework, of which 10% were double-coded to ensure coder alignment. For any disagreements which arose during the coding process, the analysts discussed the issue between themselves with the aim of coming to a decision based upon consensus. If consensus between the analysts could not be reached, the issue was referred to a third, more senior researcher who made the final decision.

A subgroup analysis of patients with acromegaly taking oral treatment compared with those on injectable treatment was conducted. Given the small sample size, the comparison was purely descriptive.

Concept saturation (19) (i.e., the point when no new information is observed in the data) was considered achieved when no new codes/concepts emerged from the next interview. Saturation was assessed for symptoms and impacts of acromegaly. Demographic or clinical data collected were analyzed using descriptive statistics.

## Results

### Demographic/clinical characteristics

A total of 15 patients from the US (mean [range] age 51.3 [42 to 64] years) were interviewed (**Table 1**). Most patients had never received surgery or radiation (n = 14, 93.3%) for acromegaly and did not suffer from other health conditions (n = 11, 73.3%). There was a balanced distribution of patients treated with injectable lanreotide (n = 6, 40.0%) and injectable octreotide (n = 4, 26.7%), and 5 patients taking oral treatment (n = 5, 33.3%). All patients receiving oral treatment were previously treated with injectable lanreotide (n = 5, 100%).

**Table 1:** Socio-demographic and clinical characteristics.

Socio-demographic/Clinical characteristics	n (%)^a^
Age (in years)	
Mean (SD) [Range]	51.3 (7.7) [42 to 64]
Gender^b^	
Female	7 (46.7%)
Male	4 (26.7%)
Preferred not to answer	4 (26.7%)
Time since diagnosis	
Less than 1 year ago	4 (26.7%)
Between 1–2 years ago	6 (40.0%)
Between 3–5 years ago	3 (20.0%)
Between 6–10 years ago	1 (6.7%)
More than 10 years ago	1 (6.7%)
Undergone surgery/radiation?^c^	
Yes, both surgery & radiation	1 (6.7%)
No	14 (93.3%)
Ethnicity/Race	
White	6 (40.0%)
Hispanic or Latino	4 (26.7%)
Black or African American	4 (26.7%)
Native Hawaiian or Other Pacific Islander	1 (6.7%)
Education level	
Completed high school or GED	4 (26.7%)
Some college	1 (6.7%)
Associate’s degree	1 (6.7%)
Bachelor’s degree	6 (40.0%)
Post-graduate degree (e.g., Masters, PhD, MD, JD)	3 (20.0%)
Relationship status	
Single	1 (6.7%)
Married	14 (93.3%)
Working status	
Working full-time	5 (33.3%)
Working part-time	1 (6.7%)
Retired or early partial retirement	4 (26.7%)
Looking after home or family	4 (26.7%)
Unemployed - seeking work	1 (6.7%)
Other health conditions	
None	11 (73.3%)
Hypertension	2 (13.3%)
Other^d^	2 (13.3%)
Current treatment	
Lanreotide	6 (40.0%)
Injectable octreotide	4 (26.7%)
Oral octreotide	5 (33.3%)

a Sociodemographic data collected during the interview (self-reported).

b Collected at screening (self-reported).

c Patients were asked ‘Have you had surgery or radiation as treatment for your acromegaly?’ during the interview and were presented with the following response options to choose from: Yes, radiation; Yes, surgery; Yes, both; No (self-reported).

d Other conditions reported were claustrophobia and anxiety.

SD, standard deviation; GED, General Educational Development; PhD, Doctor of Philosophy, MD, Doctor of medicine, JD, Doctor of law.

### Concept saturation

Saturation was assessed: all 17 symptoms were reported by the 14^th^ patient. For the 32 impacts reported, the final impact was not reported until the 15^th^ patient, but >95% were reported by the 14^th^ patient (n = 31/32, 96.9%). The saturation grids can be found in the **Supplementary Files**.

### Symptoms and disease burden

Patients most frequently reported physical changes/swelling (n = 12, 80.0%), fatigue/tiredness (n = 11, 73.3%), and excessive sweating (n = 11, 73.3%) as symptoms of their disease (**Table 2**). Physical changes were described as swelling of certain body areas, such as their hands or feet, with few patients mentioning these were permanent (n = 3, 20.0%), while others noticed an intermittent frequency (n = 2, 13.3%) or in specific situations such as when on their feet for a long time (n = 1, 6.7%). Severity of symptoms varied, and on a scale from 0 (not at all severe) to 10 (extremely severe), some patients provided ratings that ranged from 3 to 7 (mean = 5.7, n = 6). Fatigue was described as feelings of tiredness and reduced energy levels, with some patients experiencing it occasionally or infrequently (n = 3, 20.0%), while others felt it constantly or almost every day (n = 3, 20.0%), lasting from a minimum of a few hours to half a day or a couple of days (n = 3, 20.0%). On a scale from 0 (not at all severe) to 10 (extremely severe), some patients reported severity ratings between 4 and 7 (mean = 4.8, n = 4). Excessive sweating was described as sweating profusely or more than another person doing the same activity, with some patients reporting it happened occasionally (n = 2, 13.3%) or every day (n = 1, 6.7%). Patients added that this would last for half a day (n = 1, 6.7%) or mainly occurring during the night (n = 1, 6.7%). Severity varied among patients and ranged from 3 to 8 (mean = 5.0, n = 4), although three patients noted that this symptom seemed to decrease in intensity as they started receiving treatment (n = 3, 20.0%). Other less common symptoms reported by at least two patients were headaches (n = 4, 26.7%), sleeping problems (n = 4, 26.7%), weight gain (n = 3, 20.0%), and pain (n = 3, 20.0%).

**Table 2:** Symptoms reported by patients with acromegaly.

Currently experienced symptoms	n (%)^a^	Illustrative Quotes
Physical changes/swelling	12 (80.0%)	*“My hands have grown. They just seem bigger than normal. They seem almost swollen.”* (Patient 7)
Fatigue/tiredness/lack of energy	11 (73.3%)	*“I just started getting tired quicker than before, so I guess fatigue, excess fatigue.”* (Patient 9)
Excessive sweating	11 (73.3%)	*“I would sweat. If me and you were doing the same thing like raking leaves, […] you might sweat a little bit and I would sweat a lot.”* (Patient 4)
Headaches	4 (26.7%)	*“Headaches, currently I would say I experience them a handful of times a month […].”* (Patient 1)
Sleeping problems	4 (26.7%)	*“Oh, trouble sleeping? Yes, yes. […]. ”* (Patient 12)
Weight gain	3 (20.0%)	*“I’ve gained a little bit of weight.”* (Patient 10)
Pain	3 (20.0%)	*“Well, I do have, yeah, some pain in the hands, particularly I feel it in the thumb area and the index finger like if - say, I’m buttoning buttons on shirts. After I’ve gone down a couple of buttons it starts to cramp up […].”* (Patient 7)
Deeper voice / voice changes	1 (6.7%)	*“My voice got a little bit deeper and it’s raspy and it wasn’t this - used to be raspy like this before.”* (Patient 4)
Abnormal menstrual cycle	1 (6.7%)	*“My periods have been probably for the past five years a little bit irregular so I’m thinking okay maybe I’m like early, way early pre-menopause, probably the last five years.”* (Patient 1)
Heart racing / Light headedness	1 (6.7%)	*“I was lightheaded. I felt like my heart was just racing, like it was just going to pop out of my chest.”* (Patient 15)
Temperature control issues	1 (6.7%)	*“There’s times where sometimes I’m cold and other people are warm. Sometimes I’m warm and other people will say it’s a little chilly, but I’m warmer.”* (Patient 14)
Bloating	1 (6.7%)	*“I notice sometimes when I eat certain things I’m more bloated than other times. But […] before this, I was not bloated at all.”* (Patient 3)

a Symptoms are not mutually exclusive as patients could report more than one initial symptom of their condition, so numbers/percentages may not add up to 100%.

When asked, patients reported their most bothersome symptom (n>1) was excessive sweating (n = 5, 33.3%), followed by fatigue/tiredness/lack of energy (n = 4, 26.7%), physical changes/swelling (n = 3, 20.0%) and headaches (n = 2, 13.3%).

Patients reported impacts on their social life, emotional health and overall appearance. In particular, patients described a reduction in social activities (n = 6, 40.0%), increased anxiety (n = 4, 26.7%), embarrassment over sweating (n = 4, 26.7%) and changes to clothing due to swelling (n = 4, 26.7%) as the most frequently reported impacts (**Table 3**).

**Table 3:** Impacts reported by patients with acromegaly.

Impact domain	Reported impact	n (%)^a^	Illustrative Quotes
Social life /relationship with others	Does not socialize as much	6 (40.0%)	*“I don’t go out as often anymore, and when I’m around others, yeah, I kind of like to check myself. Yeah, it bothers me a little.”* (Patient 10)
	Supported by family	2 (13.3%)	*“[…] my closer family, they’ve been my rock, they have been. Always positive with me, always wanting to get me to go somewhere to do things. Go on their little concert trips and travel. They’ve been good with me.”* (Patient 14)
	No impact on social life ^b^	4 (26.7%)	*“No, I wouldn’t say so, no, it really doesn’t have an effect on my social life.”* (Patient 7)
Overall appearance / Feeling ashamed	Embarrassed/concerned of sweating/odor	4 (26.7%)	*“I don’t like to meet new people though because it’s embarrassing and they kind of judge you, you know? If you sweat and it might be - it’s embarrassing.”* (Patient 5)
	Clothing/accessories adaptations due to swelling	4 (26.7%)	*“The puffiness on my shoes, I tend to get a larger size, at least a half size, because - so I started changing shoe size.”* (Patient 3)
	Clothing adaptations due to sweating	2 (13.3%)	*“I try to also things like wear jackets so you don’t see like if my shirt is soaked, or you don’t see a sweat line underneath my arms.”* (Patient 3)
Emotional health/well-being	Anxiety	4 (26.7%)	*“It [acromegaly] makes me [feel] a little anxious, sometimes I wear dark outfits so it doesn’t show, it just makes me, as I said, a little bit embarrassing, it makes me aware of my surroundings, I’ve constantly got to be checking myself and stopping what I’m doing.”* (Patient 10)
	Depression	2 (13.3%)	*“I get depressed. I get into my little shell, and that’s not good. I know it’s not good, but I can’t help it. I can’t help it, because if I hadn’t seen it, and if I hadn’t seen it first-hand on how far it can get and how it transforms a person’s body […].”* (Patient 14)
	Insecurity	2 (13.3%)	*“[…] I just feel embarrassed, then I’m insecure about it [acromegaly] and I don’t feel very girlie, but I don’t feel emotionally that my well-being is at risk. I just don’t feel good about myself sometimes.”* (Patient 5)
	Tries to stay positive and deal with things as they come	2 (13.3%)	*“I don’t ever feel down or depressed or anything about my condition. I just try to deal with it as it comes and try to embrace the good things in life and you know, try to stay positive, you know? Try to stay upbeat about the whole thing.”* (Patient 7)
Activities of daily living (ADLs)	Limits activities due to fatigue	3 (20.0%)	*“I’d say probably just being a little less active, not walking or going to the gym as much, maybe not playing as many sports with friends, maybe taking it easy on the travel, so nothing that’s life changing, but just more making some adjustments to be a little bit less active, but still frustrating.”* (Patient 9)
Feelings about and planning for the future	Worries about future / worsening of condition	3 (20.0%)	*“I’m afraid […] it’s going to kill me […] I was very active and everything, it’s just everything is changing, you know, so it’s like sometimes, you know, it’s like what if I - is it [treatment] working, you know. What’s going to happen in three to five years, you know?”* (Patient 12)
Financial issues	No or minimal financial impact ^b^	3 (20.0%)	*“I’m lucky that I had good insurance, we have good insurance. We’ve had this for many years with the company, so I’m glad that financially I was okay. This medication is just a small amount of money and I’m glad that I can afford it. This is why it’s not a worry at this time for me.”* (Patient 2)
Physical functioning	Reduced walking/physical activity	3 (20.0%)	*“[…] I can still do everything, it’s just I might be more limited in terms of the activity. I might have to take it more easy, say, from walking a couple hours before, I might cut that down to an hour, or 30 minutes in the gym instead of 45, things like that.”* (Patient 9)
Travel and mobility	Unable to/reduced travel	3 (20.0%)	*“[…] I don’t have the energy, because as you know, when you travel, you need to walk a lot, and when you travel in a nice area or a park or in the airport or someplace […] I get tired very fast, so, yes, I have to change it to the minimum […].”* (Patient 2)
	No impact on travel and mobility ^b^	3 (20.0%)	*“Travelling […]. No, it [acromegaly] doesn’t affect that, it just - I get tired sometimes or it’s sweating but I can still travel if I have to.”* (Patient 5)
Work or caring responsibilities	No impact on work or caring responsibilities ^b^	4 (26.7%)	*“****Interviewer: Okay, does it [acromegaly] have an effect on your work or caring responsibilities?*** *Patient 7: No, I wouldn’t say so.”* (Patient 7)

a Symptoms are not mutually exclusive as patients could report more than one initial symptom of their condition, so numbers/percentages may not add up to 100%.

b The “no impact” code was not captured for every domain, only when the patient was asked and specifically responded that they did not experience an impact on this domain.

NB: The following impacts were reported by one patient only (n = 1, 6.7%): Social life: more aware of condition when meeting new people, difficult to share diagnosis with friends, condition made them distant from their friends; Emotional health and wellbeing: frustration, concerned as saw how condition affected friend, avoiding activities due to mental health; Overall appearance/feeling ashamed: appearance changes with swelling, takes better care of hands; Travel and mobility: adjustments to travel to make it easier; Activities of daily living: limits activities due to swelling, difficulty concentrating due to sweating, carrying objects awkward due to bigger hands, extreme change of lifestyle, need to wear men’s deodorant, no impact on ADLs; Feelings about/planning for the future: careful planning of events/activities; Physical functioning: unable to go walking/do physical activities; Financial issues: higher copayments; Other impacts: does less overall as more focused on health now.

#### Comparison between patients on oral treatment and injectable treatment

Patients on oral treatment (n = 5) reported being impacted socially (n = 4, 80.0%), emotionally (n = 3, 60.0%), and by their appearance/feeling ashamed (n = 4, 80.0%). Patients on injectable treatment (n = 5) reported impacts in similar domains; social (n = 3, 30.0%) and emotional (n = 5, 50.0%) impacts, as well as feelings about their appearance/feeling ashamed (n = 4, 40.0%).

### Treatment burden

Treatment aspects which frustrated patients included preparation, travel and waiting times at the clinic (n = 10, 66.7%), the general unpleasantness of getting an injection or having associated blood tests (n = 6, 40.0%), and the monthly frequency of the injections (n = 3, 20.0%). When probed further, some patients (n = 4, 26.7%) reported that monthly treatment was too frequent, while two patients described feeling fortunate that treatment was not more frequent (n = 2, 13.3%).

**Figure 1:**
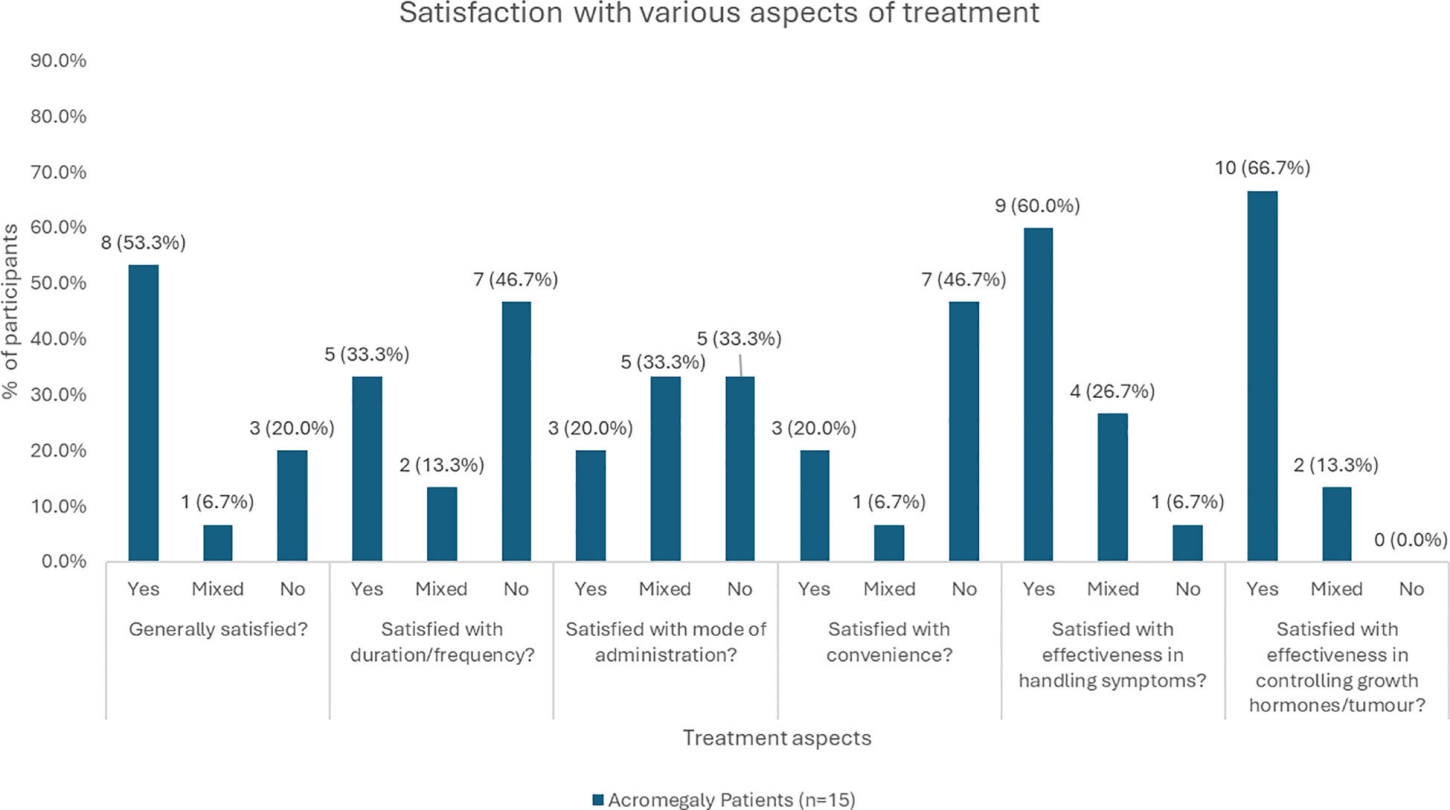
Distribution of responses in terms of satisfaction with various treatment aspects. *Not all patients provided a response to each treatment aspect, or the response may be unclear, so numbers/percentages may not add up to 100%.

In terms of satisfaction with current treatment (**Figure 1**), most patients (n = 8, 53.3%) were generally satisfied. Dissatisfaction (n = 3, 20.0%) was primarily due to the need to travel to the clinic (n = 2, 13.3%) or dislike of injections/needles (n = 1, 6.7%). While some patients (n = 5, 33.3%) were satisfied with the current duration/frequency, the majority (n = 7, 46.7%) felt it was too frequent. Similarly, only a minority (n=3, 20.0%) were satisfied with injections as the mode of administration, while some (n = 5, 33.3%) expressed dislike for injections, seeing needles, or finding them painful/uncomfortable. In terms of general convenience of treatment, only three patients (20.0%) were satisfied, while most were dissatisfied due to the need to travel to the clinic (n = 5, 33.3%), the monthly frequency of treatment (n = 2, 13.3%), or the need to undress in the clinic (n =1, 6.7%). Treatment effectiveness was highly rated, with patients reporting that it reduced symptoms such as sweating, swelling, headaches, and fatigue (n=9, 60.0%), and successfully controlled their GH levels (n=10, 66.7%).

When asked about the aspects they disliked most about their treatment, the injection/needles (n = 5, 33.3%), the need to attend the clinic (n = 4, 26.7%), and the monthly frequency (n = 3, 20.0%) were most often mentioned. The treatment aspects patients most wished to change were treatment frequency (n = 5, 33.3%), mode of administration (n = 2, 13.3%), and location of administration (i.e., at home, n = 2, 13.3%).

The most frequently reported treatment impacts were the inability to travel/plan trips due to regular treatment for acromegaly (n = 5, 33.3%), having to rest or limit activities after the injection (n = 3, 20.0%), and having to take time off for treatment (n = 3, 20.0%) (**Table 4**). Few patients (n = 2, 13.3%) reported impacts limited to the treatment days or no impact at all (n = 1, 6.7%).

**Table 4:** Impacts of treatment reported by patients with acromegaly.

Domain	Impacts	n (%)^a^	Illustrative Quotes
Travel and mobility	Unable to travel due to treatment	5 (33.3%)	*“Well, the thing is, every month I’ve got to come and get a stupid shot [monthly injection], so if I really want to go travel for like a long period of time, it’s hard to do […].”* (Patient 11)
Activities of daily living	Avoids/unable to do activities for rest of the day	3 (20.0%)	*“[…] I wasn’t prohibited from doing a whole lot of things, I just chose not to do anything strenuous or any heavy lifting or any strenuous physical work that particular day [of the injection] […].”* (Patient 10)
	No impacts on activities of daily living^b^	3 (20.0%)	*“I wouldn’t say it [treatment] affects my daily life at all. I don’t have any crazy side effects or anything. It’s just a matter of having to go in and do it.” (Patient 7)*
Emotional health/wellbeing	Anxiety	2 (13.3%)	“*[…] I think I have anxiety from it, [treatment appointments] yeah, and frustration […] I guess driving there and then putting myself through, why is this good, what the purpose is and then I’m fine.”* (Patient 6)
Social life/relationships with others	Supportive partner/family	2 (13.3%)	*“No, you know, the people that really know about it [treatment] are close family and maybe a couple of friends, but my wife’s been really supportive through the whole thing, and it’s been manageable, so I haven’t really had any major issues socially or anything like that.”* (Patient 7)
	No impact, but I plan around my treatment days	2 (13.3%)	*“I would say no [impact on social life]. It’s just one day so I just plan things around it [monthly injection].”* (Patient 5)
Work or caring responsibilities	Took time off for treatment	3 (20.0%)	*“[…] I would need to take the day off [for monthly injection] because I was super exhausted, as well. I didn’t have any energy to work, and if I did work, my boss would have thought that I’m not really doing the job correctly and he would get upset, so I would request if I could take the day off […].”* (Patient 11)
	Flexible work enables treatment	2 (13.3%)	*“Usually, I’ll take like a few hours off [for the monthly injection]. I work from home, so I don’t necessarily take the day off, but I’ll either not go in the office and work from home.” (Patient 9)*
Financial issues	Parking charges are expensive	2 (13.3%)	*“I have to do parking. They don’t validate the building, they don’t validate the parking, so I try to - it’s a little expensive these days, parking in their building is very expensive, so I try to find parking in the street.”* (Patient 2) *“No [financial impact of monthly injection], because we have pretty good insurance.”* (Patient 3)
	No financial impact ^b^	7 (26.7%)	
Other impacts	Only impacts patient on the day of treatment	2 (13.3%)	*“The treatment doesn’t impact my daily life it’s just the day I have to go [for monthly injection] that it does.”* (Patient 5)
	Impacts life but happy to prioritize treatment over other activities	2 (13.3%)	*“It’s [monthly injection] not every day or once a week or something, and I know it’s very important for my health, so that’s No. 1 in my routine and my daily work, so I keep it. I try to be on time. I try to be organizing that […] I take it very seriously.”* (Patient 2)

a Symptoms are not mutually exclusive as patients could report more than one initial symptom of their condition, so numbers/percentages may not add up to 100%.

b The “no impact” code was not captured for every domain, only when the patient was asked and specifically responded that they did not experience an impact on this domain.

NB: Impacts which were reported by one patient (n = 1,6.7%) were: Activities of daily living; tries to incorporate it into daily life, needs to allocate time for appointments, treatment days have changed lifestyle. Emotional health/wellbeing: avoids seeing it as a burden/tries to stay strong, treatments are emotional/must be mentally strong, felt guilty for interfering with friends schedules, felt it was a job to attend clinic each month, unhappy with treatment. Physical functioning: can’t do as much physically due to fatigue. Social life: no impact on social life. Work and caring responsibilities: not applicable as does not work, no impact on work. Feelings about and planning for the future: no impact on feelings about and planning for the future. Financial issues: not specified.

#### Comparison between patients on oral and injectable treatment

The main reason for patients to change from injection to oral treatment (n = 5, 100.0%) was because of the convenience of taking the medication at home (n = 4, 80.0%), although others also mentioned not having to travel to the clinic (n = 2, 40.0%) and the convenience of the mode of administration (n = 2, 40.0%). The decision to change treatment was shared between the patient and their healthcare professional (HCP) in most cases (n = 2, 40.0%), but was also driven solely by the HCP (n = 1, 20.0%) or by the patient (n = 1, 20.0%).

Patients on oral treatment highlighted several positive aspects compared to their previous injectable treatment. These included increased convenience for administration and blood testing (n = 3, 60.0%), reduced travel impacts (n = 2, 40.0%) and facilitated adherence through the use of daily reminders (n = 1, 20.0%). An advantage was the elimination of injection/needles (n = 2,40.0%) and injection site soreness (n = 2, 40.0%). Oral treatment also became part of a daily routine (n = 1, 20.0%) and maintaining control of symptoms (n = 1, 20.0%).

The negative aspects were described as the need/difficulty to remember to take the treatment at the required times/frequency (n = 2, 40.0%) and an increased concern over catching illnesses due to possible treatment interferences (n = 1, 20.0%). However, two patients did not report any negative aspects of their treatment (n = 2, 40.0%).

### Treatment preferences

Considering future treatments and factors involved in making decisions about new treatments, patients consistently prioritized side effects/safety (n = 12, 80.0%) and treatment effectiveness (n = 9, 60.0%). Other key factors included cost/insurance coverage (n = 5, 33.3%), frequency (n = 5, 33.3%), convenience/ease of use (n = 3, 20.0%), and mode of administration/invasiveness (n = 3, 20.0%).

When asked about side effects of new treatments, acceptability varied widely. Acceptable side effects included nausea/vomiting (from mild to severe; n = 4, 26.7%), headaches (n = 2, 13.3%), or mild symptoms such as stomach pain or rash (n = 2, 13.3.%). Notably, nausea/vomiting was also the most frequently mentioned unacceptable side effect (n = 8, 53.3%). Other unacceptable side-effects included major health consequences (e.g., heart conditions, kidney failure, hospitalization n = 4, 26.7%), diarrhea (n = 2, 13.3%) and loss of appetite (n = 2, 13.3%).

**Figure 2:**
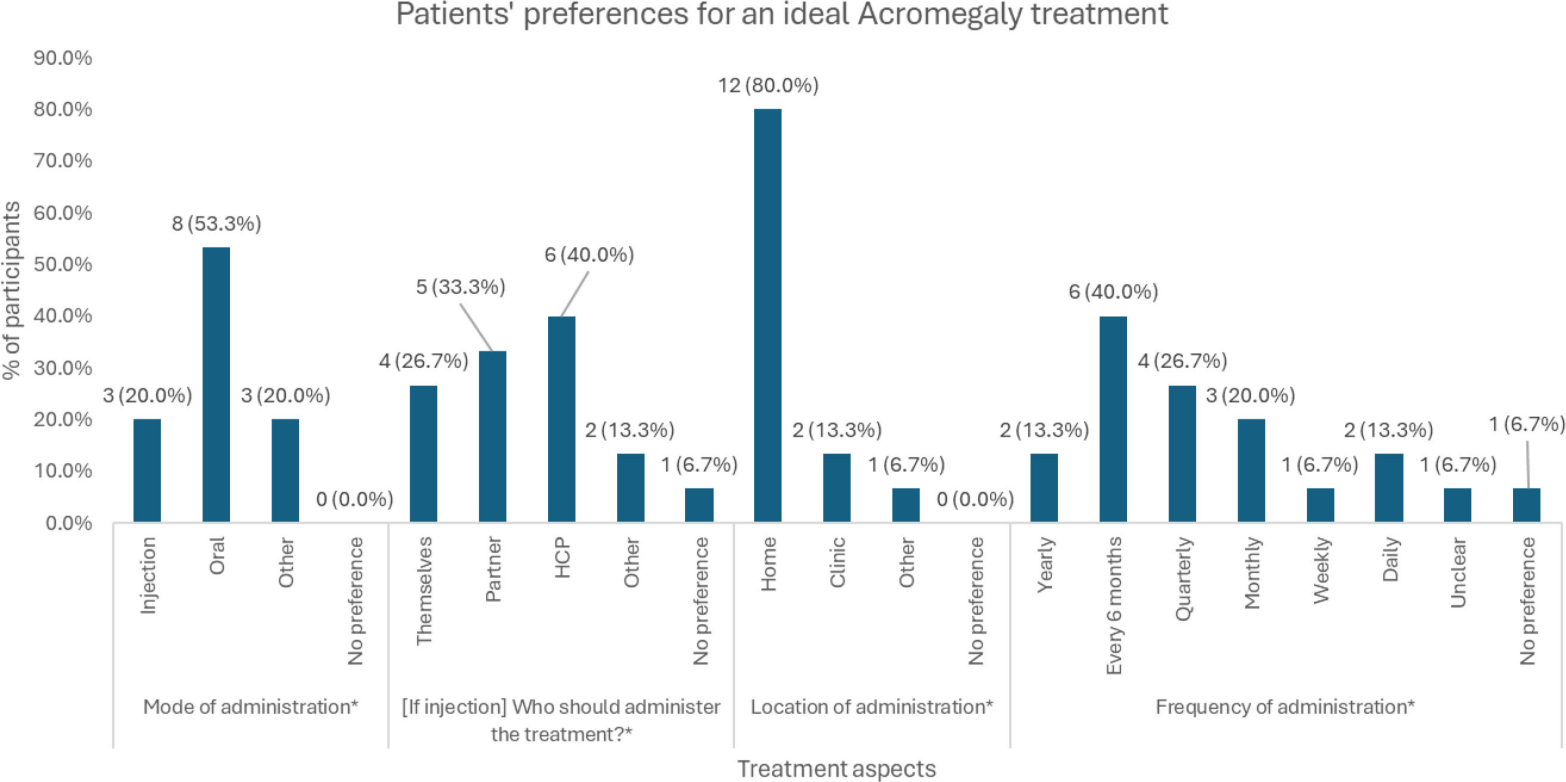
Distribution of patients’ preferences on various aspects of an ideal acromegaly treatment. * Patients could provide multiple preferences, therefore percentages may not add up to 100%. ** These frequencies of administration were preferred only if the mode of administration was a pill. HCP = Healthcare Professional.

When asked about an ideal treatment (**Figure 2**), most patients preferred treatments that were delivered at home (n = 12, 80.0%), ideally in oral form (n = 8, 53.3%). A few patients mentioned preference for an injection (n = 3, 20.0%) or other modes of administration (n = 3, 20.0%). If the treatment was an injection, this could be administered by their HCP (n = 6, 40.0%), a partner (n = 5, 33.3%), or themselves (n = 4, 26.7%). In terms of treatment frequency, a less frequent administration was preferred, with “every 6 months” being the most popular frequency (n = 6, 40.0%). If it was a pill, daily (n = 2, 13.3%) or weekly (n = 1, 6.7%) frequency was preferred.

Patients also evaluated a three-monthly injection. Almost half (n = 7, 46.7%) of the total sample (n = 15) preferred it over their current monthly treatment (**Table 5**).

**Table 5:** Preferences for three-monthly injection vs. current treatment reported by patients with acromegaly.

Preference for three-monthly injection vs. current treatment^a^	n (%)
Preference for three monthly injections	7 (46.7%)
Preference for three monthly injections only if more effective than current treatment	2 (13.3%)
Unsure on three-monthly injection preference – would depend on effectiveness and if HCP endorses it	1 (6.7%)
Preference for current treatment	0 (0.0%)

Not all patients provided a response to each treatment aspect, or the response may be unclear, so numbers/percentages may not add up to 100%.

a Monthly injection for patients on injectable treatment or daily oral treatment taken twice daily for patients on oral treatment.

Conversely, when patients on monthly injectable treatment were presented with a second hypothetical treatment (i.e., a daily oral pill), most patients preferred their current treatment (n = 8, 80.0%) over the hypothetical daily pill, citing concerns over forgetting to take pills general (n = 2, 20.0%), general dislike of pills (n = 1, 10.0%), or potential doubling of side effects (n = 1, 10.0%), while others did not share additional insights (n = 4, 40.0%) (**Table 6**).

**Table 6:** Preferences for daily tablet vs. current treatment reported by patients with acromegaly.

Preference for daily tablet vs. current treatment^a^	n (%)
Preference for current treatment	8 (80.0%)
Preference for daily tablet	2 (20.0%)

Not all patients provided a response to each treatment aspect, or the response may be unclear, so numbers/percentages may not add up to 100%.

a Monthly injection for patients on injectable treatment. Asked to patients on injectable treatment only.

#### Group differences between oral and injectable treatment preferences

There were no substantial differences between the two groups in terms of most important aspects of consideration; both prioritized side effects/safety (oral: n = 4, 80.0% and injectable: n = 8, 80.0%) and treatment effectiveness (oral: n = 3, 60.0% and injectable: n = 6, 60.0%). However, differences emerged in the ideal mode of administration. Patients on oral treatment were most likely to describe a preference for oral administration (n = 3, 60.0%) or other methods (e.g., transdermal patch) (n = 1, 20.0%). The injectable group had mixed preferences, with some preferring oral treatment (n = 5, 50.0%), but others preferring injections (n = 3, 30.0%) or alternative modes (e.g., inhaler) (n = 1, 20.0%). Both groups showed a strong preference for treatments that could be administered at home (oral: n = 3, 60.0%, injectable: n = 9, 90.0%). If injections were necessary, the group on oral octreotide favored self or partner-administration (n = 2, 40.0.%). In contrast, the group with injection had mixed preferences among self (n = 2, 20.0%), partner (n = 3, 30.0%) or HCP administration (n = 5, 50.0%). Both groups had similar views on acceptable and unacceptable side effects.

Regarding treatment frequency, patients on oral treatment valued a three-monthly injection and noted this would be a better frequency (n = 3, 60.0%); only one patient (n = 1, 20.0%) noted a negative aspect of needing to set reminders. Patients taking oral treatment also noted a negative aspect of taking a daily pill (n = 2, 40.0%) as the need to remember to take the pill, which was also reported as a treatment impact.

### Disease impact model

**Figure 3:**
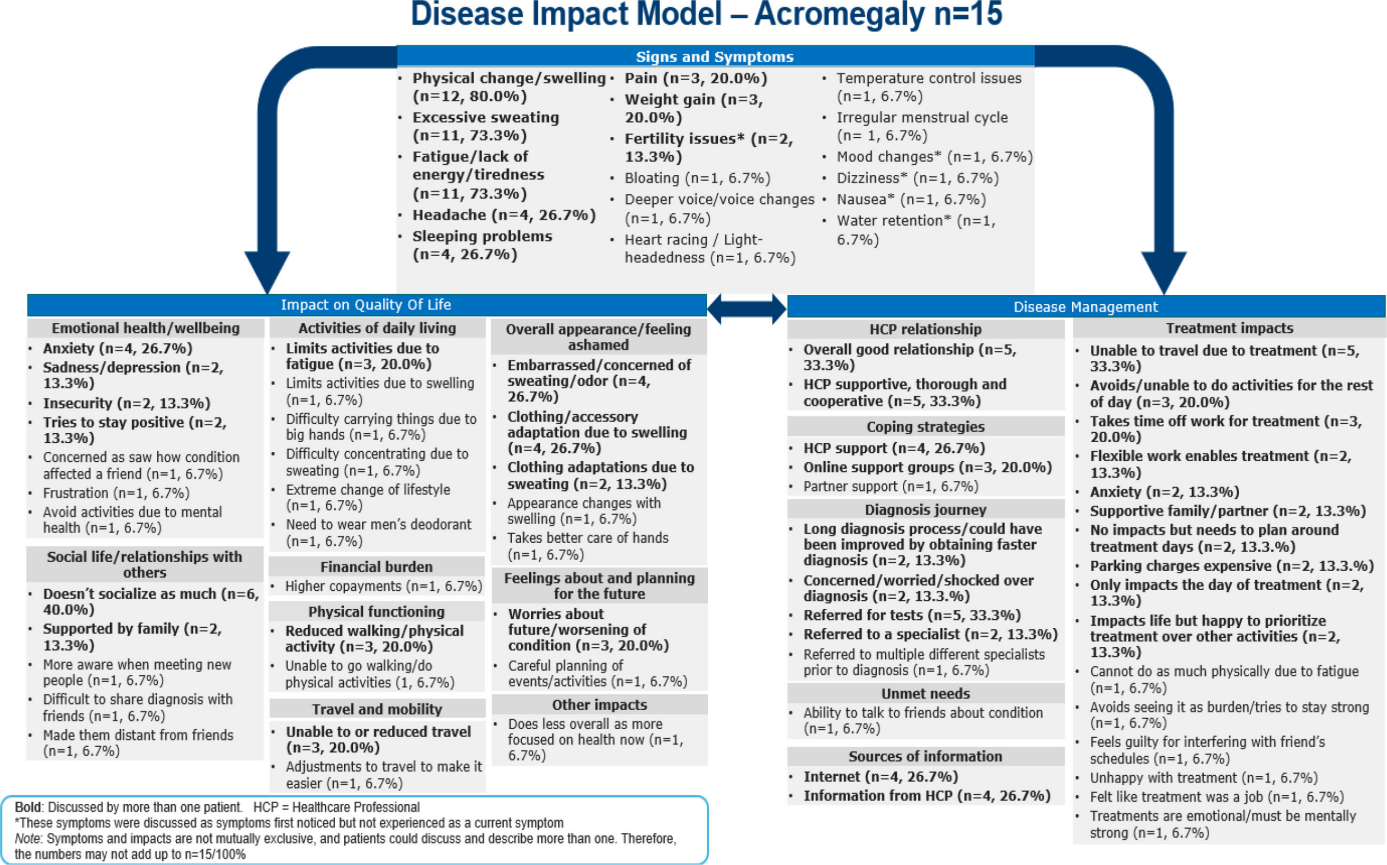
Disease impact model.

Concepts which emerged from analysis of the interview transcripts (n = 15) were used to develop a disease impact model outlining signs and symptoms impacting the patients’ health related quality of life (HRQoL) and aspects related to disease management (**Figure 3**).

## Discussion

This study investigated the experiences of patients with acromegaly in relation to their disease and treatment burden and explored their treatment preferences in terms of ideal treatment attributes, as well as the value of a sustained release treatment. To date, there is little published evidence on the burden of treatment associated with monthly injections (and associated hospital visits) or on patient preference in this area.

When exploring disease burden, the wide-ranging and complex impacts of acromegaly on patients found in this study were consistent with findings from other studies in acromegaly, such as time expenditure for managing illness and the impacts of physical changes/swelling, as well as patients’ mental wellbeing, emotional wellbeing and social functioning (14, 18–21).

Patients in the current study discussed the impact of treatment, explaining their treatment impacted them mostly only on the days they received their injection and indicating that less frequent administration could potentially reduce treatment burden. This was caused by various aspects related to their treatment days, such as travelling to the clinic, clinic waiting time and the impact on their travel/plan trips, which were all exacerbated by the monthly treatment frequency. However, if the frequency of health checks was kept to a monthly frequency, as reported by several patients, then the benefits of this reduced frequency may be lost or minimized, particularly if the health checks involved blood tests. Further confirming these results, when discussing their treatment preferences regarding various aspects of treatment, most patients suggested preferring a home-administered treatment with a longer lasting duration of effect than the current monthly treatment, as these could help reduce some of the treatment-related impacts reported in this current study. This finding was also reflected in patients’ choices when discussing a hypothetical three-monthly injection compared to their current monthly injection.

Patients demonstrated a preference for oral treatments that could be administered at home, when asked about their preferences around various treatment aspects. However, when comparing a hypothetical oral treatment administered twice a day to their current treatment, 80% of patients on injectable treatment preferred their current treatment over a daily oral, due to the possibility of forgetting daily doses and the potential side effects they may experience. This confirmed previous published findings which showed preference for a home-administered injectable SSA against either the injection administered at hospital (22) or an oral treatment administered at home (23). This demonstrates a preference for patients to take ownership of their treatment administration and not needing to rely on their HCP or to travel for treatment. However, the current study adds to these previous findings by highlighting the possible limitations of a daily oral treatment, which could affect patients’ decision to switch to an oral treatment.

Patients taking oral treatment provided insight into why they chose to switch to the daily oral treatment from the monthly injectable treatment. The preference for treatments that could be administered orally at home compared to intravenously at a clinic have been shown in other patient populations, such as patients with cancer, diabetes, and rheumatoid arthritis (24–27). However, similarly to the current findings, many of these studies also stated that treatment needed to provide equal or superior effectiveness with a similar safety/side effects profile to be preferable to patients. Safety/side effects and treatment efficacy were the most important aspects when considering new treatments, similarly to what was shown by Seo et al. in patients on SSA treatment (28).

### Strengths and limitations

The current study benefited from the completion of a literature review prior to conducting interviews, identifying gaps in the evidence aided the development of the interview guide. The literature review showed that there were no previous studies which assessed the treatment burden of SSA injections and treatment preferences in patients with acromegaly. Furthermore, there were few qualitative studies evaluating the disease and symptom burden in this population, highlighting the valuable contribution of this research. The sample recruited for this study showed some diversity in terms of ethnicity, age, and education, which ensured a diverse range of patients’ experiences were captured and explored. A robust analysis approach, including double coding a proportion of transcripts, ensured accuracy and consistency between analysts.

Nonetheless, this study has some limitations. First, the study included a small sample size of patients on oral treatment compared to injectable treatment, making comparison of these samples difficult. Additionally, all patients were recruited from the US, which could limit the applicability of the results to other countries and healthcare systems. Furthermore, recall bias may have been a factor when patients on oral treatment were asked to discuss their previous injectable treatment, which they were no longer taking at the time of the interview, or individuals for whom it had been a long time since initial diagnosis. Finally, a high proportion of patients in the study did not undergo surgery or radiation prior to receiving injectable SSA treatment. In addition, all patients in the oral-treatment subgroup had recently received injectable SSA before switching to oral therapy. This may have shaped their experiences and preferences, which may differ from those of patients with prior surgery or radiation experience or who had never received injectable SSA.

Ultimately when asking patients to discuss their preferences on aspects of treatment, patients were given options for a limited number of aspects, such as administration method and frequency of administration. As shown in the results of this study, there were multiple factors which could contribute to decision making, such as efficacy, safety, and the HCPs’ own opinions and understanding of the treatment. Therefore, it is possible that the preferences stated may differ from decisions patients would make in real-life situations when offered a new treatment, where patients are dealing with a more complex risk-benefit trade-off. This limitation could be addressed in a future discrete choice experiment methodology investigating the most important treatment aspects identified in this study and how they would influence patients in their treatment decision-making process.

## Conclusion

This qualitative study provided valuable insights and contributions to the evidence for disease burden as well as treatment experience, burden, and preferences for patients with acromegaly. Patients described a wide range of symptoms and the associated impacts on their lives. The results indicated a high burden of treatment associated with monthly SSA injections and patients demonstrated a strong preference for less frequently administered treatments, with specific preferences shown towards three- and six-monthly injections over the current monthly standard of treatment for injectable SSA and also home-administered treatments. High importance was placed on the efficacy and safety profile of any new treatment. These insights suggest that transitioning to less frequent dosing schedules has the potential to lead to improved HRQoL and better clinical outcomes, though these prospective benefits would require further longitudinal validation in larger cohorts.

## Data Availability

The original contributions presented in the study are included in the article/**Supplementary Material**. Further inquiries can be directed to the corresponding author.
