# Supplementary material for: Disease burden, treatment experiences and preferences in patients with acromegaly: a qualitative study
Source: Front Endocrinol (Lausanne). 2026 Mar 5;17:1733510. doi: 10.3389/fendo.2026.1733510 (PMC12999441; doi:10.3389/fendo.2026.1733510)
Supplement: Supplementary file 2 [file DataSheet1.pdf]

Supplemental material

Article title: Disease Burden, Treatment Experiences and Preferences in Patients with Acromegaly: A Qualitative Study

Journal name: Frontiers in Endocrinology

Author names and affiliations: Jennifer Quinn[1], Andrea De Palma[2], Rebecca McKeown[3], Rocco Adiutori[4], Charlotte E. Kosmas[3], Isabelle Petit[1]

[1] Debiopharm International SA, Lausanne, Switzerland

[2] Erasmus School of Health Policy & Management, Erasmus Rotterdam University, Rotterdam, The Netherlands

[3] ICON plc, Insights, Evidence and Value, Reading, United Kingdom

[4] ICON plc, Insights, Evidence and Value, Milan, Italy

Corresponding author email address: isabelle.delattrepetit@debiopharm.com

Supplementary material

Targeted literature review results

The literature review retrieved 290 references, and 11 articles were selected for data extraction after the screening, which included data from patients and health care providers obtained by interviews, surveys and focus groups (Supplementary Figure 1*)*. The studies were conducted in different regions of Europe, United States, and Brazil. Signs and symptoms relevant to patients with acromegaly were identified, but also key domains impacting on health-related quality of life (HRQoL), disease management and treatment burden and preferences. The data extracted aided the development of the interview guide.

Targeted literature review: Search strategy

The literature review was performed using Ovid platform, including Medline and APA PsycInfo databases, using the following search strategy

1. Disease terms: exp Acromegaly/ or (acromegaly or "hypersecretion syndrome, somatotropin (acromegaly)" or "hypersecretion syndromes, somatotropin (acromegaly)" or "inappropriate gh secretion syndrome (acromegaly)" or "inappropriate growth hormone secretion syndrome (acromegaly)" or "somatotropin hypersecretion syndrome (acromegaly)" or "somatotropin hypersecretion syndromes (acromegaly)" or "syndrome, somatotropin hypersecretion (acromegaly)" or "syndromes, somatotropin hypersecretion (acromegaly)").mp.
2. Qualitative terms: exp mixed methods research/ or exp Focus group interview/ or exp Focus groups/ or exp Interviews as topic/ or exp Interview, Psychological/ or exp Qualitative methods/ or exp Qualitative research/ or exp Cognitive interview/ or exp Self Report/ or exp Narrative analysis/ or exp Interview/ or exp Personal Narrative/ or exp Personal Narratives as Topic/ or exp Narration/ or (Qualitative or Grounded or Phenomenological or Focus group* or thematic analysis or conceptual framework or personal experience or verbatim or Semi-structured interview* or semistructured interview* or structured interview* or in-depth interview* or Qualitative interview* or exploratory interview* or individual interview* or face-to-face interview* or Narrative* or Narration or Conceptual model* or Concept elicitation).mp.
3. Concept terms: exp Patient Satisfaction/ or exp Patient Preference/ or exp "Treatment Adherence and Compliance"/ or (Treatment satisfaction or Treatment preference or Patient satisfaction or Patient preference or "ease of use" or compliance or Complain* or acceptance or acceptability convenien* or client treatment preference or patient experience or compliance or non-compliance or adherence or non-adherence or treatment burden or "Treatment Adherence and Compliance").mp.
4. Limits: presence of abstract, english language, publication year (from 2002 to 2022), humans
5. #1 AND (#2 OR #3) AND #4

Search performed on October 20th 2022, retrieving 290 references.

/: Thesaurus term

exp: exploded term

mp: multi-purpose fields in Medline (title, abstract, original title, name of substance word, subject heading word, protocol supplementary concept word, rare disease supplementary concept word, unique identifier) and APA PsycInfo (title, abstract, heading word, table of contents, key concepts, original title, tests & measures)

PRISMA flowchart

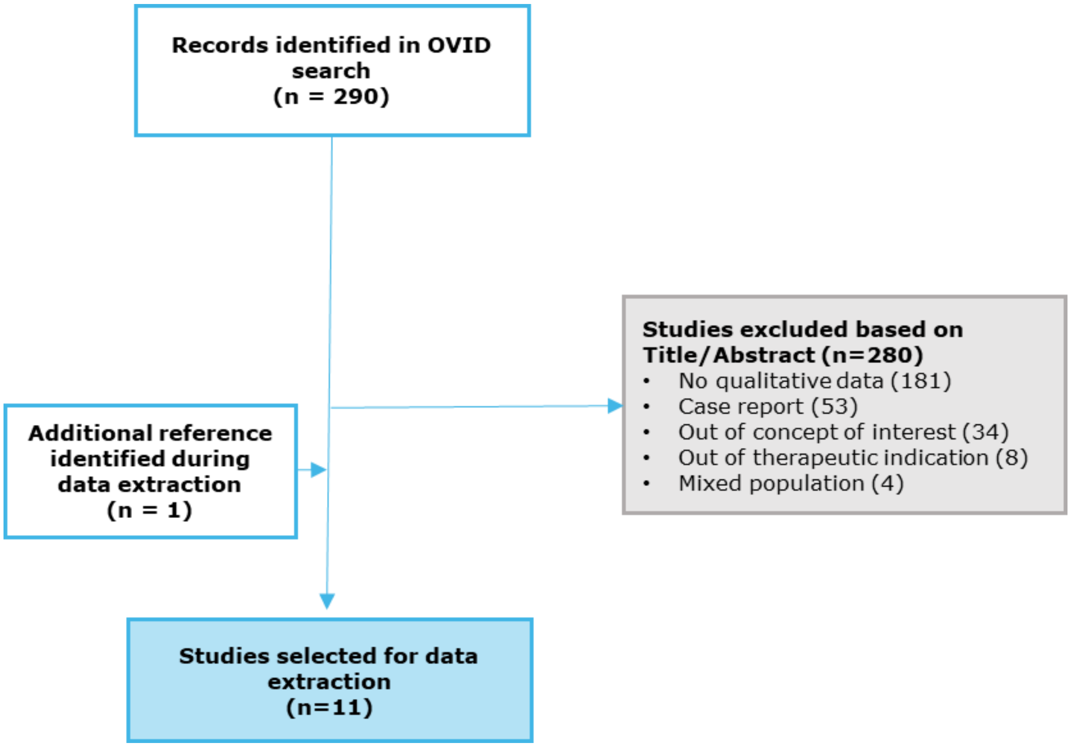

Supplementary Figure 1. PRISMA flowchart of the targeted literature review
