# Supplementary material for: Disease burden, treatment experiences and preferences in patients with acromegaly: a qualitative study
Source: Front Endocrinol (Lausanne). 2026 Mar 5;17:1733510. doi: 10.3389/fendo.2026.1733510 (PMC12999441; doi:10.3389/fendo.2026.1733510)
Supplement: Supplementary file 3 [file DataSheet2.pdf]

Symptoms	Acromegaly Interviews														
**Patient ID by order of interview**	**P1**	**P2**	**P3**	**P4**	**P5**	**P6**	**P7**	**P8**	**P9**	**P10**	**P11**	**P12**	**P13**	**P14**	**P15**
Physical changes / swelling*	x	x	x	x	x	x		x	x	x	x	x	x	x	x
Fatigue / tiredness / lack of energy*	x		x	x	x	x	x	x	x	x		x	x	x	x
Excessive sweating*		x		x		x	x		x	x	x	x	x	x	x
Headaches*	x	x		x						x					
Sleeping problems*					x		x		x	x		x			
Weight gain*	x		x				x								x
Pain*				x				x	x	x					
Deeper voice / voice changes*		x													
Abnormal menstrual cycle	x														
Heart racing / Light headedness*													x		
Temperature control issues														x	
Bloating												x			
Fertility issues*			x												x
Mood changes*										x					
Dizziness*	x														
Nausea*	x														
Water retention*												x			
**New symptoms applied**	7	2	1	1	1	0	0	0	0	1	0	2	1	1	0
**Cumulative # of symptoms applied (%)**	7 (41.2%)	9 (52.9%)	10 (58.8%)	11 (64.7%)	12 (70.6%)	12 (70.6%)	12 (70.6%)	12 (70.6%)	12 (70.6%)	13 (76.5%)	13 (76.5%)	15 (88.2%)	16 (94.1%)	17 (100.0%)	17 (100.0%)

Notes:
x indicates that symptom was reported by the patient
Cells highlighted in green indicate the first occurrence of a new symptom
* indicates symptoms that were also reported as initial symptoms prior to diagnosis

Impacts		Acromegaly Interviews														
**Patient ID by order of interview**		**P1**	**P2**	**P3**	**P4**	**P5**	**P6**	**P7**	**P8**	**P9**	**P10**	**P11**	**P12**	**P13**	**P14**	**P15**
**Impact domain**	**Specific impact**															
Social life / relationship with others	Does not socialise as much					x	x	x			x		x		x	
	Supported by family													x	x	
	More aware when meeting new people									x						
	Difficult to share diagnosis with friends														x	
	Made them distant from their friends														x	
Emotional health / well-being	Anxiety			x				x			x		x			
	Depression										x				x	
	Insecurity							x		x						
	Tries to stay positive and deal with things as they come				x											x
	Concerned as they saw how condition affected friend														x	
	Frustration					x										
	Avoids doing activities due to mental impact												x			
Overall appearance / feeling ashamed	Embarrassed / concerned of sweating / odour							x		x		x	x			

Impacts		Acromegaly Interviews														
**Patient ID by order of interview**		**P1**	**P2**	**P3**	**P4**	**P5**	**P6**	**P7**	**P8**	**P9**	**P10**	**P11**	**P12**	**P13**	**P14**	**P15**
**Impact domain**	Specific impact															
	Clothing /accessories adaptations due to swelling					x							x	x	x	
	Clothing adaptations due to sweating							x					x			
	Appearance changing with swelling										x					
	Takes better care of hands													x		
Travel and mobility	Unable or reduced travel						x		x						x	
	Adjustments to travel to make it easier					x										
Activities of daily living (ADL)	Limits activities due to fatigue	x			x	x										
	Limits activities due to swelling														x	
	Difficulty concentrating due to sweating							x								
	Carrying objects awkward due to bigger hands					x										
	Extreme change of lifestyle												x			
	Need to wear men's deodorant												x			
Feelings about and planning for the future	Worries about future / worsening of condition				x						x				x	
	Careful planning of events / activities	x														

Impacts		Acromegaly Interviews														
**Patient ID by order of interview**		**P1**	**P2**	**P3**	**P4**	**P5**	**P6**	**P7**	**P8**	**P9**	**P10**	**P11**	**P12**	**P13**	**P14**	**P15**
**Impact domain**	Specific impact															
Physical functioning	Reduced walking / physical activity					x					x					x
	Need to take breaks during an activity / tires easily	x														
	Unable to go walking / do physical activities															x
Financial issues	Higher copayments										x					
Other impacts	Does less as more focussed on health now								x							
**Total # impacts per patient**		3	0	1	3	7	2	6	2	3	7	1	8	3	10	3
**Total # impacts**																
**New impacts applied**		3	0	1	2	6	1	4	1	1	3	0	3	2	4	1
**Cumulative # of impacts applied (%)**		3 (9.4%)	3 (9.4%)	4 (12.5%)	6 (18.8%)	12 (37.5%)	13 (40.6%)	17 (53.1%)	18 (56.3%)	19 (59.4%)	22 (68.8%)	22 (68.8%)	25 (78.1%)	27 (84.4%)	31 (96.9%)	32 (100.0%)

Notes:
x indicates that impact was reported by the patient
Cells highlighted in green indicate the first occurrence of a new impact
